# Single-particle electroluminescence of CsPbBr_3_ perovskite nanocrystals reveals particle-selective recombination and blinking as key efficiency factors

**DOI:** 10.1038/s41467-019-12512-y

**Published:** 2019-10-03

**Authors:** Dharmendar Kumar Sharma, Shuzo Hirata, Martin Vacha

**Affiliations:** 1 Department of Materials Science and Engineering, Tokyo Institute of Technology, Ookayama 2-12-1-S8-44, Meguro-ku, Tokyo, 152-8552 Japan; 2 Department of Chemistry, Maulana Azad National Institute of Technology, Bhopal, 462-003 India; 3 Department of Engineering Science and Engineering, The University of Electro Communications, 1-5-1 Chofugaoka, Chofu, Tokyo, 182-8585 Japan

**Keywords:** Nanoparticles, Quantum dots

## Abstract

Halide perovskites nanocrystals (NCs) are being explored as promising materials for optoelectronic applications, such as light-emitting devices or lasers. However, electroluminescence devices prepared from such NCs have long suffered from low efficiency and there has been no systematic study on the nanoscale origin of the poor efficiencies. Here, we use single-particle spectroscopy to compare electroluminescence and photoluminescence on the level of individual NCs of the perovskite CsPbBr_3_. The NCs form aggregates in a conducting matrix used as an emission layer in an electroluminescence device. In electroluminescence, only a small fraction of the NCs within the aggregate is emitting as a result of efficient charge migration, accumulation and selective recombination on larger NCs, leading to pronounced blinking and decreased efficiency. Under the condition of comparable excitation rates in both electroluminescence and photoluminescence, the intrinsic quantum yield in electroluminescence is on average 0.36 of that in photoluminescence.

## Abstract (web summary)

Halide perovskite nanocrystals show high photoluminescence (PL) but their electroluminescence (EL) devices suffered from low external quantum efficiency. Here Sharma *et al*. study both individual and statistical PL and EL behaviors of these nanocrystals and reveal the origin of the lower EL efficiency.

## Introduction

Organic–inorganic or all-inorganic lead halide perovskites have recently re-emerged as one of the most promising semiconducting materials for applications in optoelectronic devices. Their application in solar cells has in a short period of time revolutionized the field of alternative non-silicon photovoltaic devices^1–3^. Metal halide perovskites posses remarkable optoelectronic properties, such as direct bandgap, wide-range bandgap tunability, large absorption cross-section, and narrow photoluminescence (PL) linewidth^4^. Together with their low cost, feasibility for scale-up synthesis, solution processability, and compatibility with existing optoelectronic device components, these properties make metal halide perovskites a feasible alternative to other semiconducting materials for a range of optoelectronic applications, including light-emitting applications for displays and lighting, lasers, and memory devices^5–9^. Applications for light-emitting diodes (LED) have been attempted even before the current research boom but realization of electroluminescence (EL) required cryogenic temperatures^10^. The first LEDs based on solution-processed organic–inorganic halide perovskites achieved external quantum efficiencies (EQE) of less than 1%^11^. One direction towards the improvement of perovskite LED device performance is the use of confinement of charges in perovskite nanocrystals (NCs)^12,13^. Confinement of the highly mobile charges into nanometer-scale structures results in high PL quantum yield (PLQY), radiative lifetime on the order of nanoseconds^14^, and a resulting high brightness. At the same time, liquid suspensions of the NCs can be handled in a similar way as solutions of organic dyes or colloidal quantum dots (QD) in the device manufacture. The first attempts to use CsPbX_3_ (X = Cl, Br, I) perovskite NCs as emitters in LEDs resulted in EQE of 0.12%^15^. The performance has been since improved greatly by ligand exchange and ligand density control^16–19^, cross-linking^20,21^, surface defect passivation^22–25^, Ag doping^25^, in-situ growth^26^, suppression of Auger non-radiative recombination^27^, or ion exchange^28^. These efforts resulted recently in fabrication of LEDs with EQE exceeding 15%^28,29^. Despite this fast progress in device engineering, there has been no systematic study on the nanoscale physical origin of the poor efficiencies.

In this work, we attempt at different approach in studying the phenomenon of EL in halide perovskite NCs by performing nanoscale characterization of EL devices. Specifically, we use single-particle detection and spectroscopy to characterize individual NCs of CsPbBr_3_ in PL and EL. The single-molecule method has been successfully used in studying a range of problems in materials science including organic optoelectronic materials^30–33^. The fact that single-molecule spectroscopy has the power to uncover heterogeneities and dynamics of physical parameters that are otherwise hidden in ensemble averages has helped, e.g., to reveal the inhomogeneous nature of the green emission band in PL and EL of single chains of the conjugated polymer polyfluorene and contributed to the assignment of its origin^34^. The method was also used to study single-molecule EL of small-molecule organometallic complexes^35^ or of CdSe QD^36^.

Here, we study single-particle PL and EL of NCs of the perovskite CsPbBr_3_ surface-passivated with oleylamine ligands. The EL is studied in devices which use as an emitting layer a film of conducting polymer polyvinylcarbazole (PVK) mixed with PBD, in which the CsPbBr_3_ NCs are dispersed at very low concentrations. The EL is compared with PL on the same NCs within the devices in terms of emission site localization, emission intermittency (blinking), and emission spectra, and with PL of single CsPbBr_3_ NCs in pure PVK film. The main finding is that there is only a small fraction of NCs which are active in the EL process but the photophysical behavior of these EL-active NCs is comparable to that in PL.

## Results

### Imaging and blinking of perovskite NCs in PL and EL

**Fig. 1:**
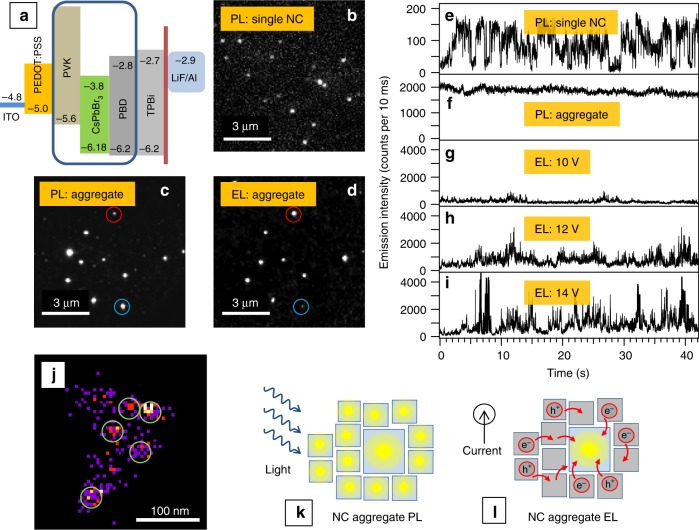
Imaging and blinking of NC aggregates in PL and EL. **a** Scheme of the EL device structure, together with the relevant energy levels. **b** Microscopic PL image of single NCs of CsPbBr_3_ in PVK matrix. **c** Microscopic PL image of aggregates of CsPbBr_3_ NCs in EL device. **d** Microscopic EL image (at a bias of 14 V) of the same NCs aggregates as in **c**. In **c** and **d**, the blue circles denote an example of an aggregate which shows stronger PL, red circles an aggregate which shows stronger EL. **e** Intensity time trace of PL of single CsPbBr_3_ NCs in PVK matrix. **f** Intensity time trace of PL of aggregates of CsPbBr_3_ NCs in EL device; intensity time trace of EL of aggregates of CsPbBr_3_ NCs at **g** 10; **h** 12; and **i** 14 V. **j** Super-resolution EL image obtained by analysis of a series of microscopic EL images of aggregates of CsPbBr_3_ NCs at 14 V over the time of 60 s. **k** Schematic illustration of PL from an aggregate showing independent emission from individual NCs. **l** Schematic illustration of EL from an aggregate showing charge migration (red arrows) and emission from the largest NC which works as a trap

Electroluminescence of CsPbBr_3_ NCs was studied in a device of the structure ITO/PEDOT:PSS/PVK: CsPbBr_3_:PBD/TPBi/LiF/Al, as shown in Fig. 1a. The emitting layer is a spin-coated film of PVK and PBD into which CsPbBr_3_ NCs are doped at different concentrations. The synthesized CsPbBr_3_ NCs have approximately cube-like shapes, average size of 16 ± 5 nm (Supplementary Fig. 1), and are passivated with oleic acid and oleylamine ligands. The whole device is prepared on a microscope cover glass to enable direct microscopic EL and PL observation using high numerical aperture objective lenses^34^. Details of the microscopic setup and the EL device are shown in Supplementary Fig. 2. The lowest concentration of CsPbBr_3_ NCs used corresponds to complete dispersion of individual NCs in the matrix. PL microscopic image of such sample (Fig. 1b) reveals emission from well separated and isolated single NCs which show the phenomenon of PL intensity blinking. However, devices prepared with this truly single-particle level concentration of CsPbBr_3_ NCs do not show any detectable EL under the same microscope, up to a bias of 18 V. To detect EL we gradually increased the concentration of the CsPbBr_3_ NC doping. At concentrations higher than the single-particle dispersions, the NCs do not disperse homogeneously in the matrix but rather tend to form aggregates. When reaching a certain size, such aggregates can be detected in microscopic images both in PL (Fig. 1c) and EL (Fig. 1d). The turn-on voltage for the EL images is 6 V. Figure 1c, d shows the same area of the sample and most of the NC aggregates can be identified in both excitation modes. However, the relative emission intensities can be different. Some aggregates are more intense in PL (e.g., the one denoted by blue circles) while others are more intense in EL (red circles) and some aggregates appear only in one of the images. Compared to single NCs which have been reported to exhibit strong PL blinking in PVK matrix before^37^ (such as the one shown in Fig. 1e), most of the NC aggregates do not show any blinking in PL due to the intra-aggregate ensemble averaging (Fig. 1f). We note that there are fluctuations of the PL intensity that are larger than the noise in many aggregates (Fig. 1f) but we do not classify these as blinking. Such fluctuations are most likely caused by the finite number of PL active NCs in the aggregates. On the other hand, the same aggregates exhibit strong blinking in EL, involving multiple intensity levels, as shown for three different voltages in Fig. 1g–i. The traces in Fig. 1f–i are all measured from the same aggregate.

### Aggregate size and super-resolution analysis

As seen in the microscopic images in Fig. 1c and d, the size of some of the aggregates is larger than the microscope resolution (diffraction limit). To estimate the actual aggregate size and the number of NCs involved in the emission process, we prepared samples for atomic force microscopy (AFM) and transmission electron microscopy (TEM) from solutions of comparable NC concentrations as those used to prepare the EL devices. While the TEM and AFM imaging provides correct and consistent estimate of the aggregate size in toluene, we realize that even at comparable NC concentrations the presence of the polymer matrix can affect the aggregation. Therefore, the following characterization should be taken as indicative information rather than as direct evidence of the aggregate size. AFM imaging (an example of which is shown in Supplementary Fig. 3a) was used to analyze the aggregate lateral size. The histograms in Supplementary Fig. 3b, c show a large distribution of aggregate sizes from a few tens to more than a hundred of nanometer. The TEM imaging (an example shown in Supplementary Fig. 3d) confirms this lateral size range, and shows that the aggregates are more-or-less symmetric in the 2D sample plane. The above characterization indicates that for the CsPbBr_3_ NCs to be active in EL, they have to form aggregates of the sizes of between 50 and 100 nm in diameter. Such aggregates typically contain tens to hundreds of individual NCs.

The fact that the aggregates do not blink in PL but show strong blinking in EL can result from one of two possibilities: 1. All (or majority of) the NCs emit with very low efficiencies. Because of that, each of the NCs would be dark most of the time and only occasionally emit bursts of EL photons. Averaging of these bursts over all NCs would form the intensity blinking traces (such as the ones in Fig. 1g–i). 2. The EL occurs relatively efficiently from just a few NCs and these are responsible for the blinking while the remaining ones are dark permanently. To look into these two possibilities, we analyzed the EL images using the technique of super-resolution microscopy^38^. Super-resolution microscopy is based on sub-diffraction microscopic localization of individual emitters which is achieved by 2D Gaussian fitting of their emission images. The well-established super-resolution techniques such as PALM or STORM^38^ require controlled switching of emission of a sub-ensemble of organic dye labels. Instead of the active switching we make use of the strong blinking of EL which has the form of stochastic intensity bursts occurring from the background level (Fig. 1i). Regarding the two possibilities above, in the case of all NCs emitting at the same time with very low efficiency we do not expect to observe any preferred EL locations or their changes. An example of the super-resolution analysis shown in Fig. 1j indicates the presence of several (five in this case) distinct emitting spots within the aggregate. Data collected from other aggregates (examples presented in Supplementary Fig. 4) show that there are typically three to seven emitting sites in each aggregate, the location of the sites changes in time and they emit EL consequently. This is an important result which implies that out of the tens to hundreds NCs that form an aggregate and that are all actively emissive in PL, only a few are actually emitting in the EL process. In other words, there is a selection process in place that causes only a small fraction of the CsPbBr_3_ NCs to efficiently undergo charge recombination and EL emission.

First, we exclude a possibility that the selectivity in EL is a result of non-uniformity of the device, such as roughness of the surface caused by the presence of the NC in the emitting layer or other geometrical factors. We used AFM to measure the surface quality and thickness of individual layers during the device fabrication process (Supplementary Fig. 5), and summarized the results in Supplementary Table 1. Looking specifically at the most critical layers of PVK:CsPbBr_3_:PBD and TPBi, the roughness of the surface (rms) is 0.27 and 0.51 nm, respectively, indicating no influence of the dispersed NCs on the surface structure. In this context, the current device differs from a recently reported LEDs based on submicrometer-scale perovskite structures in which surface roughness caused by the presence of the structures played important role in the device function and light extraction^39^. To further characterize possible non-uniformity of the electric field in our device we measured background EL emission originating from the PVK/PBD matrix at different locations where the NCs are absent. The background appears smooth and without any spatial intensity variations. The spatial uniformity was also confirmed by preparing an OLED device with a PVK/PBD emitting layer, i.e., without doping with perovskite NCs. Microscopic imaging of such device (Supplementary Fig. 6) shows spatially and spectrally uniform emission both upon electrical excitation and photoexcitation, and this uniformity was confirmed at different random locations in the device.

### Spectral characterization in PL and EL

**Fig. 2:**
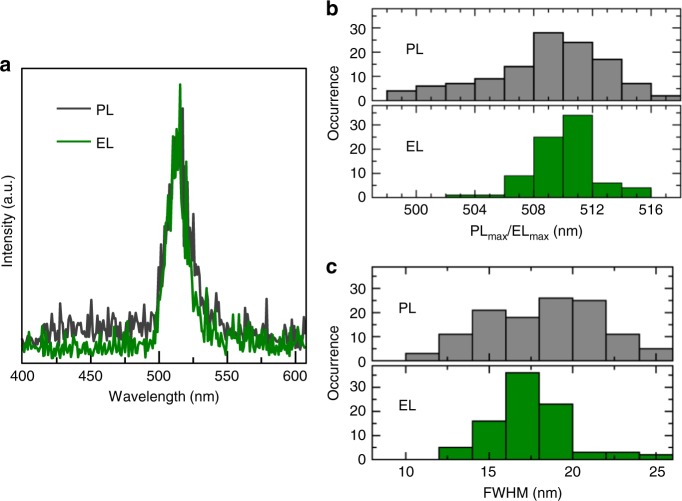
Spectral characterization in PL and EL. **a** Example of PL (black, excited at 485 nm) and EL (green, at the bias of 14 V) spectra taken from the same NC aggregate in the EL device. **b** Distribution of PL (top) and EL (bottom) spectral peak positions. **c** Distribution of PL (top) and EL (bottom) spectral linewidths (FWHM). The distributions in **b** and **c** were constructed from 80 NC aggregates in EL at the bias of 14 V and from 118 NC aggregates in PL excited at 485 nm

To get further insight into the nature of the selectivity in EL we studied emission spectra from the same aggregates in PL and EL. An example of such spectra in Fig. 2a shows that there is, in this case, little difference between the lineshape and position of the PL and EL spectra. Analysis of a statistical ensemble of NCs (118 in PL and 80 in EL) provides distributions of both spectral peaks and linewidths (full width at half maximum (FWHM)) in EL and PL. As seen in Fig. 2b, c, both distributions are narrower in EL compared to PL. Also, the maximum of the distribution of the spectral peaks in EL is slightly red-shifted compared to that in PL. The average size of the NCs in this study falls in weak confinement regime^40,41^ where moderate dependence of bandgap energy and PL spectral peak on NC size can still be expected (Supplementary Note 1, Supplementary Fig. 1d). The red shift of the EL spectral distribution thus reflects larger average size of the NCs. The narrower distribution of the linewidths in EL reflects a narrower size distribution. Overall, these results indicate that of the wide size distribution of CsPbBr_3_ NCs present in the device, only larger NCs of more uniform size are preferably active in the EL emission. The larger size is related to smaller bandgap and consequently to shallower valence band for efficient hole trapping and deeper conduction band for efficient electron trapping.

### Excitation rates and emission intensities in PL and EL

The fact that only one or a few NCs are active in EL emission can be caused by inefficient charge injection in most of the NCs or by non-radiative recombination of the charges in such NCs. As a result, the majority of the NCs would be dark and only a fraction would be emitting in EL, as observed in the super-resolution images. Another possible explanation involves electrical interactions between individual NCs in the aggregate. In such case, the charges would be initially trapped by all the NCs, but the electrons and holes would then migrate within the aggregate. The migration can be driven by the electric field and by the energy landscape of the aggregate. As mentioned above, due to the quantum confinement, larger NCs have smaller bandgap energy which shifts both the valence and conduction bands to form deeper traps for both charges. Such energy landscape would cause accumulation of the migrating charges in the deepest traps, that is, on the few largest NCs, and only these would be emitting EL. This effect of charge funneling, which has recently been observed on quasi-2D multilayer perovskites^42^ or within composition-gradient single-crystalline perovskite nanowires^43^, resembles the phenomenon of energy funneling which is often observed in multichromophoric systems such as conjugated polymers. In such systems, the exciton migration and trapping causes emission from a limited number of chromophores and results in observation of blinking even in very large systems^33,44^.

To distinguish the above two possibilities, i.e., inefficient charge injection/non-radiative recombination vs. charge migration, it is necessary to evaluate and compare the excitation (absorption) rate in PL and the charge recombination rate in EL on individual NCs. The absorption rate *n*_a_ can be calculated from the known^41^ intrinsic absorption coefficient *μ*_i_ for CsPbBr_3_ which is related to the absorption cross-section *σ* via the NC volume *V*_NC_ as *σ* = *V*_NC_*μ*i. Using the excitation intensity of 26 W cm^−2^ (at 485 nm) gives the *n*_a_ value of 1.1 × 10^7^ s^−1^. To estimate the charge recombination rate, we adopted the approach used for imaging of single organic molecules in OLED devices^45^. Briefly, we assume Langevin-type recombination^46^ in which the recombination rate *n*_r_ is given by

$$n_{\mathrm{r}} = \frac{{\pi r_{\mathrm{c}}^2}}{e}j,$$

where *j* is current density and *r*_c_ a capture radius. The capture radius can be estimated as *r*_c_ = 30 nm for the NCs in PVK (Supplementary Note 2). To determine the current density, we prepared a matrix-only device with the same structure as the one used for the single-particle EL experiments but without the perovskite NCs doped. The current density was determined from an *I*–*V* dependence (Supplementary Fig. 7) measured on this device. At 14 V, *j* = 0.08 A cm^−2^ which gives the recombination rate *n*_r_ of 1.2 × 10^7^ s^−1^. The actual recombination rate will be influenced by many factors and its correct estimation is more complex, and as such the value of *n*_r_ obtained above should be treated as an upper limit of the true rate. Even so, this value is surprisingly well comparable with the absorption rate during photoexcitation.

Since the excitation rates per single NC are comparable in PL (with 26 W cm^−2^ excitation) and in EL (at 14 V bias), it is now possible to compare the absolute values of the emission intensities from the same aggregate in PL and EL. These are plotted on the *y*-axis of Fig. 1e–i. The EL intensity trace at 14 V shows strong blinking with the maxima (ON times) reaching 3500 counts per 10 ms. The PL intensity of the aggregate shows steady emission with intensity around 2000 counts per 10 ms. Compared to these, the PL intensity trace of a single NC shows blinking with maxima levels more than an order of magnitude smaller than the aggregate. The non-blinking PL trace of the aggregate is due to simultaneous independent emission of all (or most) of the NCs, and the intensity difference between the aggregate and the single NC is consistent with tens of individual NCs emitting at the same time (Fig. 1k). The lack of blinking indicates that energy funneling by efficient exciton migration and subsequent emission from only one NC is not a dominant process in these NC aggregates. On the other hand, the emission intensity levels observed in the EL trace help to distinguish between the inefficient charge injection/non-radiative recombination versus charge migration hypotheses mentioned above. The fact that the EL intensity from one (or a few) NCs is comparable to the PL intensity from the whole aggregate excludes the possibility of non-radiative recombination on most (dark) NCs and radiative recombination on only one or a few of them. Rather, the emitting NC must be efficiently populated by the migrating charges (charge funneling) from most of the NCs within the aggregate to account for the peak EL intensity. The whole aggregate thus works as a “charge antenna” and the captured charges are accumulated on the largest NC, from where the EL takes place (Fig. 1l). We note that a limit to an optimum size of such charge antenna will originate from a saturation of the optical transition of the emitting NC due to a finite lifetime of the exciton.

### Blinking analysis

**Fig. 3:**
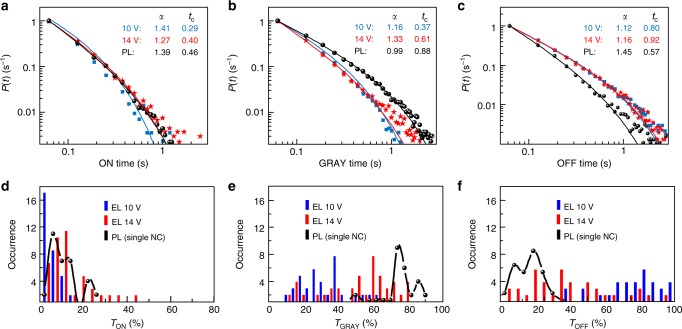
Blinking analysis of NC aggregates in EL and single NCs in PL. Probability density distributions (**a**–**c**) and distribution of fractional times (**d**–**f**) for ON (**a**, **d**), GRAY (**b**, **e**), and OFF states (**c**, **f**) for aggregates of CsPbBr_3_ NCs in EL (blue—10 V bias, red—14 V bias) and for PL of single CsPbBr_3_ NCs in PVK (black). All the probability density curves are fitted with a truncated power law equation ($P\left( t \right) = {\mathrm{A}}t^{ - \alpha }{\mathrm{e}}^{ - t/t_{\mathrm{c}}}$) and the fitting coefficient values are shown in the individual plots. A fractional time of a state reflects a percentage of the whole measurement interval over which the NC was in such state

We further look in detail at the strong EL blinking characteristics, as presented in Fig. 1g–i. The EL intensity is not changing between two distinct (ON and OFF) levels as often observed for perovskite NCs but rather shows fluctuations involving multiple intensity levels^47–52^. For the purpose of further analysis, the levels were classified into ON states (for normalized intensities above 0.7), GRAY states (for intensities between 0.25 and 0.7), and OFF states (for intensities below 0.25) (Supplementary Fig. 8). This approach has been used in our recent study on environment-induced blinking of CsPbBr_3_ NCs^37^. The ON level above 0.7 corresponds to the average ON intensity minus twice the standard deviation of noise to minimize the effect of the noise in the analysis. Similarly, the OFF level below 0.25 corresponds to the average background (OFF) plus twice the standard deviation of the noise. The blinking analysis was carried out in terms of probability density distributions and fractional times of the individual intensity levels. Since the blinking in EL originates from a single NC within the aggregate, it is instructive to compare such blinking with PL blinking of a single NC in the same environment of a conducting PVK matrix, shown in Fig. 1e. The probability density distributions *P*(*t*) of ON, GRAY and OFF times are shown in Fig. 3a–c. They can be well fit with a truncated power law function $P\left( t \right) = {\mathrm{A}}t^{ - \alpha }{\mathrm{e}}^{ - t/t_{\mathrm{c}}}$, where *α* is an exponent and *t*_c_ is a critical time^53,54^. The exponent *α* characterizes the length (duration) of a period of the respective intensity level and its decrease indicates an increasing length of the respective event. Figure 3a–c shows an example of the effect of bias voltage on the blinking dynamics by comparing the analysis results for 10 and 14 V bias. An increase in the bias increases the average duration of the ON times (the exponent *α*) and decreases the average duration of the GRAY times. The OFF times are not particularly affected by the bias. The overall fractions of the respective intensity levels within individual NC intensity traces are presented in the form of histograms in Fig. 3d–f. A fraction of, e.g., ON time gives a percentage of the whole measurement interval over which the NC was in ON state. Here, it is apparent that increasing the voltage from 10 to 14 V causes a slight increase of the ON fraction, but the main effect is the increase of GRAY time fractions (Fig. 3e) at the expense of OFF time fractions (Fig. 3f). We have previously studied the PL blinking of CsPbBr_3_ NCs in PVK under externally applied voltage^37^. We found that the effect of the conducting matrix (with HOMO level close to the NC valence band, such as PVK) and/or the effect of the external electric field lead to decrease of brightness due to the appearance of the GRAY intensity states. We assigned the ON intensity levels to emission of neutral excitons and the GRAY levels to emission of singly negatively charged excitons (negative trions)^37^. Such assignment is in broad agreement with previous work on perovskite NCs in which dim levels in two-state blinking were assigned to charged excitons^47^. It is also consistent with the extensive work done on II–VI semiconductor QD, where, e.g., a recent detailed study on CdSe nicely showed the appearance of three distinct PL intensity levels due to neutral, negatively and positively charged excitons, respectively^55^. The OFF states likely originate from differently charged species.

The above comparison of the EL and PL blinking characteristics shows that, at least in terms of the ON level duration (Fig. 3a) and ON level fraction (Fig. 3d), the truly single NC in PL and the active single NC in EL emission (especially at 14 V bias) are well comparable, that is, the neutral excitons in both processes are emitting in a similar way. The main differences are in the GRAY level duration and fractions which are larger in PL, and in the OFF level durations which are larger in EL. The OFF fractions are similar between the EL (at 14 V bias) and PL blinking. Overall, the single NCs which are selectively excited in the EL are comparable in their neutral exciton emission characteristics to single NCs in PL in the same conductive environment.

### Sequential and simultaneous PL and EL excitation and emission

**Fig. 4:**
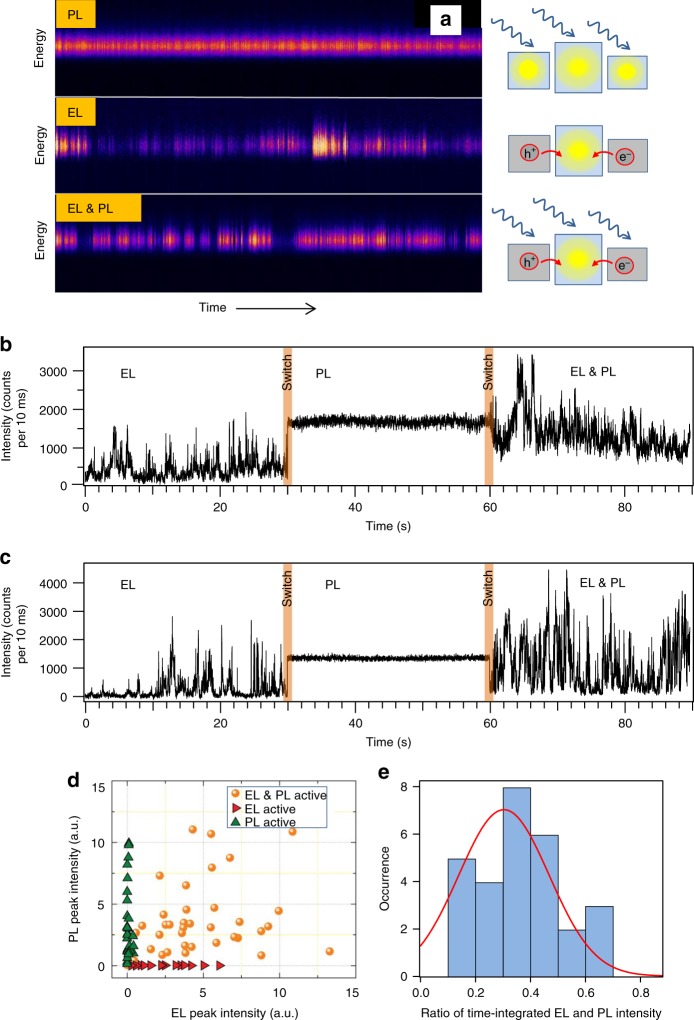
Comparison of PL and EL characteristics. **a** Two-dimensional plots showing time evolution of emission spectra from an individual CsPbBr_3_ NC aggregate, excited by light (top), charge recombination (middle), and combination of both (bottom). The scheme on the right explains the observations. **b**, **c** Time-traces of emission intensity obtained from two different single aggregates during switching of the excitation modes. PL was excited at 485 nm with 26 W cm^−2^, EL with bias of 14 V. **d** Correlation plot of 37 NCs that were active in both PL and EL (orange), in PL only (green), and in EL only (red). **e** Distribution of the ratio of EL and PL intensity, each integrated over the measurement time of 40 s

A further insight into the mechanism is provided by simultaneous excitation of the same NC aggregate by light (PL) and charge recombination (EL). The results are shown in the form of 2D spectral plots (time evolution) in Fig. 4a. In PL-only (top trace) the aggregate emits continuously, similar to the aggregate PL blinking trace in Fig. 1f. In EL-only (middle trace) the same aggregate shows multi-level blinking, similar again to the EL traces in Fig. 1g–i. When both excitation modes are applied simultaneously (bottom trace) the aggregate shows blinking with a range of intensities different from the EL-only trace. The apparent absence of blinking in PL is, as mentioned earlier, due to an averaging effect. The strong blinking in the EL-only trace reflects the charge funneling and emission from only one (or a few) NC(s) within the aggregate. Photoexcitation of an aggregate in which charges migrate over individual NCs (the simultaneous PL and EL excitation regime, bottom trace) will lead to quenching of PL at those NCs that are charged at the moment of photoexcitation. As a result, only a few NCs which are neutral during the photoexcitation, including possibly the EL-active NC(s), can be excited by light and will be emitting PL. Figure 4b, c shows examples of intensity traces from two aggregates measured during switching between the excitation regimes. While the EL and PL parts look similar to the traces in Fig. 1f–i, the simultaneous EL and PL traces resemble more the PL trace in Fig. 4b, and more the EL trace in Fig. 4c. More examples of this type of differences are shown in Supplementary Fig. 9. In both Fig. 4b and c, the simultaneous EL and PL traces show larger peak intensities than either EL or PL alone. Since the excitation rates in EL and PL are comparable in the simultaneous EL and PL traces, the shape and intensity of the traces indicate that the emission is originating both from EL and PL, with varying contribution of each in different aggregates.

As observed in the PL and EL images and intensity traces in Fig. 1c, d, f–i and Fig. 4b, c, it is interesting to note that some of the aggregates are more intense in PL while others are more intense in EL, and some aggregates even appear only in one of the images. To quantify this observation, we constructed a correlation plot of the EL and PL intensities obtained on the same aggregates (Fig. 4d). The intensity values in Fig. 4d are obtained from spectrally resolved intensity traces (such as the ones in Fig. 4a) by integrating over the area under the spectral peak and over the time of 1 s. In case of the blinking (mainly EL) traces, the time interval of 1 s is chosen over the intensity maxima (blinking ON states). Overall, there is a more-or-less positive correlation of the PL and EL intensities on the same aggregates, that is, high PL intensity generally corresponds also to high EL intensity. The same positive correlation is also observed between maxima of the PL and EL emission spectra, as shown in Supplementary Fig. 10. We assume that for those aggregates which emit strongly in PL but are absent or emit weakly in EL, the charge migration and injection efficiency is poor for most or all the NCs in the aggregate and as a result not even a single NC is emissive in EL. The opposite case of aggregates appearing only in the EL images may be caused by multiple charging of the NCs in the whole aggregate—such aggregate would be dark in the PL, but charge migration could be still going on and injection during the EL process could lead to partial reduction of some of the charges, resulting in singly-charged NCs and observable EL emission.

Apart from the above comparison of the peak intensities, we also analyzed the integrated intensities in PL and EL over the measurement time of 40 s. These integrated intensities reflect the total number of photons emitted during the time interval and account for the blinking ON, GRAY, and OFF levels. Since for the excitation conditions used (26 W cm^−2^ at 485 nm for PL and 14 V for EL) the excitation rates are comparable in PL and EL, the differences in the integrated intensities (number of emitted photons) should directly reflect differences between the intrinsic quantum yields (QY) in PL and EL. The results of the analysis are shown in the form of a histogram of the ratio of integrated EL to integrated PL intensity (Fig. 4e). The histogram spans a range between 0.12 and 0.66, and the average EL/PL ratio is 0.36. The large width of the histogram reflects the inherent errors of the quantum yields. Such errors originate from errors in the excitation rates and errors in the emission intensity. The error in the absorption rate in PL originates mainly from the size distribution of the NCs, leading to a distribution in the intrinsic absorption coefficient at the excitation wavelength. The error in the recombination rate in EL is more complex and originates mainly from the fact that the current is measured in a reference device, from several assumptions in the model describing the recombination, and is also contributed by the size distribution. The errors in the emission rates in both PL and EL arise from the effects of local environment, size distributions, but the biggest factor is the presence and numbers of singly and multiply charges NCs within the aggregates. All these factors contribute to the resulting distribution of the EL/PL ratio. We therefore evaluated the standard deviation of the distribution as 0.11, and consider this an overall experimental error of the EL/PL value.

## Discussion

We investigated on single-particle level the PL and EL of NCs of the perovskite CsPbBr_3_ surface-passivated with oleic acid and oleylamine ligands. Aggregates consisting of tens to hundreds NCs do not show any blinking in PL due to simultaneous emission of all NCs. In EL, only a small fraction of the NCs within the aggregate is active due to charge migration and selective charge recombination, leading to pronounced blinking. The EL-active NCs are characterized by narrow size distribution and larger size. The larger size results in lower bandgap energy and causes the larger NCs to function as traps where the charges migrating over the whole aggregate accumulate and recombine. The EL-active single NCs at a specific bias voltage show similar emission and blinking properties in the ON states as single NCs in PL. Analysis of the numbers of emitted photons shows that assuming comparable excitation rates for the NC aggregates in PL and EL, the intrinsic ELQY is on average 0.36 of the PLQY. We have previously measured the PLQY of CsPbBr_3_ NCs dispersed in PVK on ensemble level and found a value of 0.3 for the excitation wavelength of 485 nm^37^. This leads to the ELQY value of 10.8%. Assuming further a typical outcoupling efficiency of a light-emitting device of 20% gives the external ELQY of about 2%. This value is consistent with previous earlier reports on EL of CsPbX_3_ perovskite NCs with the same ligand passivation^7,8^ and may explain the poor EL efficiencies of this type of material. The present study points a way towards efficient nanoscale characterization of EL of halide perovskite materials, and will enable, e.g., screening of the effect of surface passivation and ligands on the charge injection in individual NCs.

In conclusion, we studied PL and EL on single aggregates composed of tens to hundreds of NCs of the perovskite CsPbBr_3_. Energy landscape of the aggregate caused by size distribution of the NCs leads to funneling of charges towards and EL from the largest NCs. The blinking phenomenon occurring on these EL-emitting NCs is the main origin of the decrease of EL quantum efficiency.

## Methods

### Synthesis of CsPbBr_3_

CsPbBr_3_ NCs have been synthesized by a slight modification^37^ of the method developed by Protesescu et al.^56^. Briefly, the cesium precursor was synthesized using dried and degassed Cs_2_CO_3_ (107.6 mg), oleic acid (0.30 ml), and 1-octadecene (ODE, 5 ml). A clear solution of Cs-oleate in ODE was obtained at 140 °C and kept at 100 °C. The PbBr_2_ precursor was prepared by mixing dried and degassed PbBr_2_ in 7.5 ml ODE (107.3 mg), 0.5 ml oleic acid, 0.5 ml oleylamine (technical grade 70%) at 120 °C under N_2_ environment. The solution was heated upto 225 °C and 0.6 ml of Cs-oleate, pre-heated at 100 °C, was quickly injected into this reaction mixture. When the reaction mixture turned yellow-green (after a few second) the reaction was stopped by rapid cooling in an ice bath. The synthesized CsPbBr_3_ NCs were precipitated in a mixture of *tert*-butanol and ODE (5 ml each) at room temperature and then centrifuged at 8000 r.p.m. for 15 min. Finally, the synthesized NCs were re-dispersed in toluene and stored at 4 °C in dark for further experiments.

### Bulk spectroscopic characterization

Ensemble spectroscopic characterization was performed on CsPbB_3_ dispersed in toluene by UV–Vis absorption (V760, Jasco) and PL (Quantaurus-QY, Hamamatsu Photonics) spectrometers.

### Sample preparation for single-particle PL imaging and spectroscopy

For single-particle PL measurements, nM-order suspensions of CsPbBr_3_ in a toluene solution of a conductive polymer poly (9-vinylcarbazole), PVK (M_n_ approx. 25,000 to 50,000; MERCK/Sigma Aldrich) were prepared by sonicating for 5–10 min. This solution was spin cast at ~1500 r.p.m. (60 s) onto a freshly cleaned micro cover glass (24 × 24 mm, 0.15 mm, Matsunami). All the samples of CsPbBr_3_ were prepared in ambient environment and dried under vacuum before mounting on the microscope.

### Device preparation for single-particle EL imaging and spectroscopy

The EL devices were prepared on microscope cover slides (22 × 22 mm, 0.15 mm thickness) on which an ITO electrode (56.2 nm thickness, 100 Ω sq^−1^; Geomatec) has been custom-deposited by sputtering. The ITO layer was etched to form stripes 5 mm in width. The substrates were pre-cleaned by sonicating in acetone and 1-propanol. A 58 nm hole-injection layer of poly(3,4ethylenedioxythiophene) poly(styrenesulfonate) (PEDOT:PSS, Clevios P AI4083, H. C. Starck) was spin coated (4000 r.p.m., 40 s) on the ITO substrate and baked at 200 °C for 90 min. The active layer is a mixture of PVK and 2-(4-biphenylyl)-5-(4-tert-butylphenyl)-1,3,4-oxadiazole (PBD, B8378-5G; Merck) at a ratio of 7:3, doped with the appropriate concentration of the suspensions of CsPbBr_3_. The layer was spin-coated from chlorobenzene to form a film of 52 nm thickness. The sample was placed in a vacuum chamber where a 15 nm electron-transport layer of 2,2′,2″-(1,3,5-Benzinetriyl)-tris(1-phenyl-1-H-benzimidazole) (TPBi), 1 nm layer of LiF and an Al electrode (200 nm) were subsequently evaporated. The Al electrode was deposited in form of stripes 5 mm in widths, orthogonal to the ITO stripes. The device element area was 0.25 cm^2^. The device was used for experiments immediately.

### Sample preparation for TEM and AFM characterization

Samples for TEM imaging were prepared by diluting freshly synthesized CsPbBr_3_ sample with toluene to about 10 nM and sonicating for 5 min. A drop of the solution was placed on a carbon-coated grid and left to dry prior to mounting on the TEM sample holder. For AFM imaging, toluene solution with the same concentration of CsPbBr_3_ as the one used for TEM was spin-coated on clean mica substrate and used for the experiment.

### Single-particle PL and EL setup

Imaging and spectroscopy have been performed on a multimodal fluorescence microscope in epifluorescence mode^37^. Briefly, Olympus (IX71) inverted optical microscope with oil immersion objective lens (Olympus, UPlan FLN ×100/1.3 NA, Oil) was used for the EL and PL imaging. In PL, the samples were excited with a pulsed 485 nm diode laser (LDH-PC-375/485, PicoQuant) and the emission was collected using electron-multiplying (EM) CCD camera (iXon, Andor Technology) after passing an imaging spectrograph (Bunkou Keiki, CLP-50, 0.5 nm resolution) and appropriate optical filters. In EL, a dc bias voltage was provided and controlled by a multi-meter (Keithley, Tektronics) and a function generator. The EL signal was detected in the same way as the PL signal. The acquisition times were typically varied between 10 and 50 ms per frame for the blinking and 100 and 200 ms per frame for the spectra. All measurements were performed in air at room temperature.

### Data analyses

Single-particle imaging and spectroscopy data were analyzed using ImageJ2 (NIH), OriginPro 2016, and MATLAB 2017. PL images were background corrected for excitation field modulations using a rolling ball algorithm. The intensity time trajectories from well-separated emission spot (3 × 3 pixel) were obtained using “z-profile” in ImageJ and data were exported in ASCII format for further analysis. PL spectral profiles from spectrally resolved images were obtained by integrating three pixels along vertical (position) direction and an average background of the same dimension was subtracted to obtain the PL spectrum. The spectrograph was calibrated using several laser lines. The spectral information such as peak positions and FWHM for each particle was obtained by fitting with a Gaussian function. The time evolution of the spectra is presented by making a montage of the movie frames of the individual particle in the same sequences of their recording. Super-resolution analysis was carried out using the ThunderSTORM^57^ or SMLocalizer^58^ plugin in ImageJ software. The analysis was applied consequently to individual frames in a series of 1200 EL images (each taken with 50 ms exposure time) and the resulting positions of individual emitters at different times were overlaid to form the composite image, such as the one in Fig. 2e. Any possible stage drift during the acquisition of the series was compensated. The resolution in the super-resolution analysis is approximately 10 nm, as evaluated by line profile fit of the super-resolved images.

## Supplementary information

Supplementary Information

## Data Availability

The data that support the findings of this study are available from the corresponding authors upon reasonable request.
