# Supplementary material for: Single-particle electroluminescence of CsPbBr3 perovskite nanocrystals reveals particle-selective recombination and blinking as key efficiency factors
Source: Nat Commun. 2019 Oct 3;10:4499. doi: 10.1038/s41467-019-12512-y (PMC6776502; doi:10.1038/s41467-019-12512-y)
Supplement: Supplementary file 1 — Supplementary Information [file 41467_2019_12512_MOESM1_ESM.pdf]

Supplementary Information

Single-particle electroluminescence of $CsPbBr_3$ perovskite nanocrystals reveals particle-selective recombination and blinking as key efficiency factors

Dharmendar Kumar Sharma[1,2], Shuzo Hirata[3], Martin Vacha[1]

[1]Department of Materials Science and Engineering, Tokyo Institute of Technology, Ookayama 2-12-1-S8-44, Meguro-ku, Tokyo 152-8552, Japan
[2]Department of Chemistry, Maulana Azad National Institute of Technology, Bhopal 462-003, India
[3]Department of Engineering Science and Engineering, The University of Electro Communications, 1-5-1 Chofugaoka, Chofu, Tokyo 182-8585, Japan

Correspondence and requests for materials should be addressed to D.S.K (dksiitb@gmail.com) and M.V. (vacha.m.aa@m.titech.ac.jp)

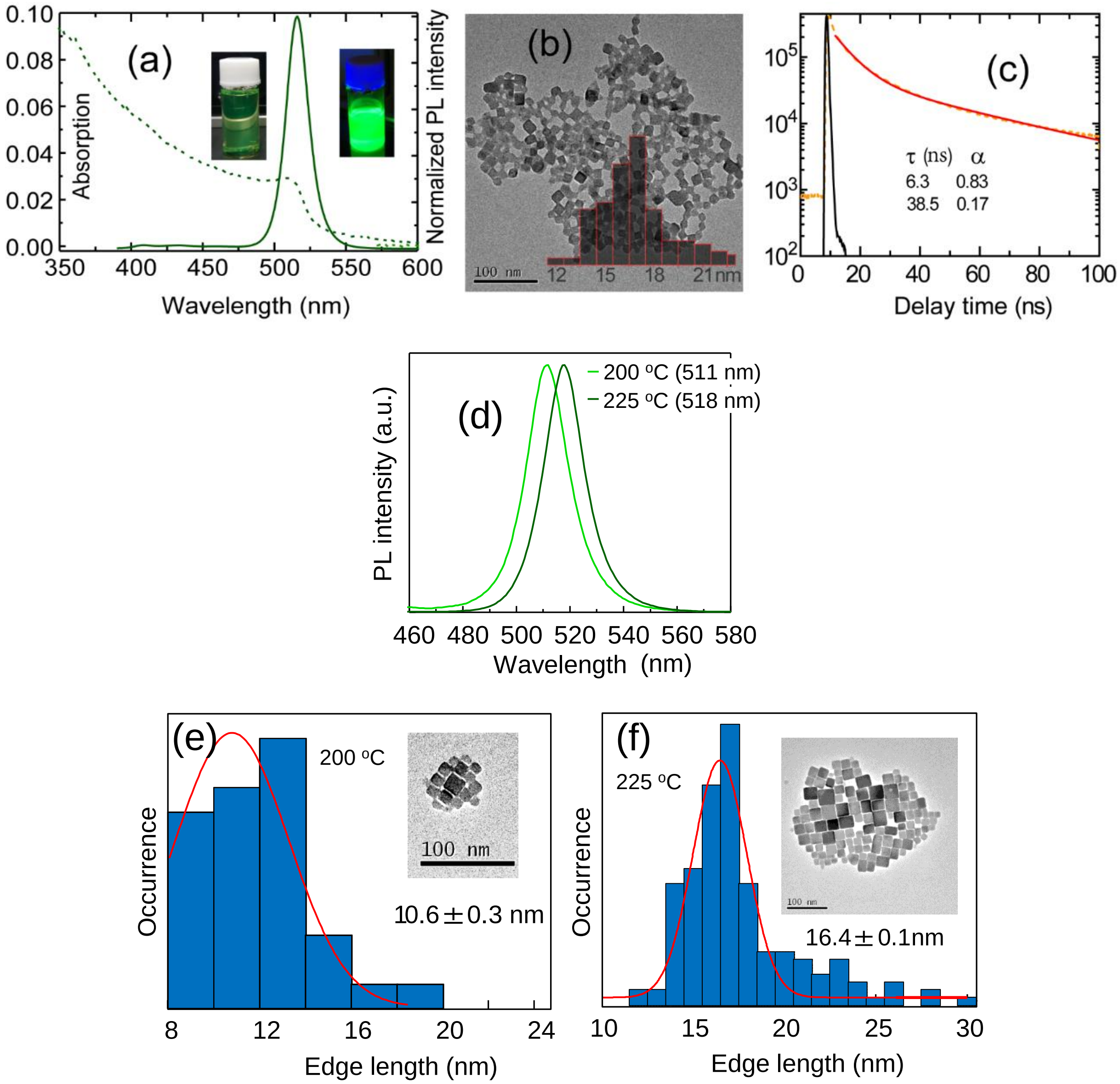

Supplementary Figure 1. (a) UV-visible absorption spectra (dotted line) and PL emission at 375 nm excitation. Inset shows color images of $CsPbBr_3$ without and with UV lamp radiation. (b) TEM- image and size distribution plot on top, shows cubic shape of $CsPbBr_3$ crystals of size ~ 16 nm (range 12 - 20 nm). (c) PL intensity decay curves of $CsPbBr_3$ NCs at 375 nm excitation and their corresponding double exponential fitting curves. (d) PL spectra of of $CsPbBr_3$ NCs synthesized at 200 ^{o}C and 225 ^{o}C, and their corresponding size distributions (e) and (f).

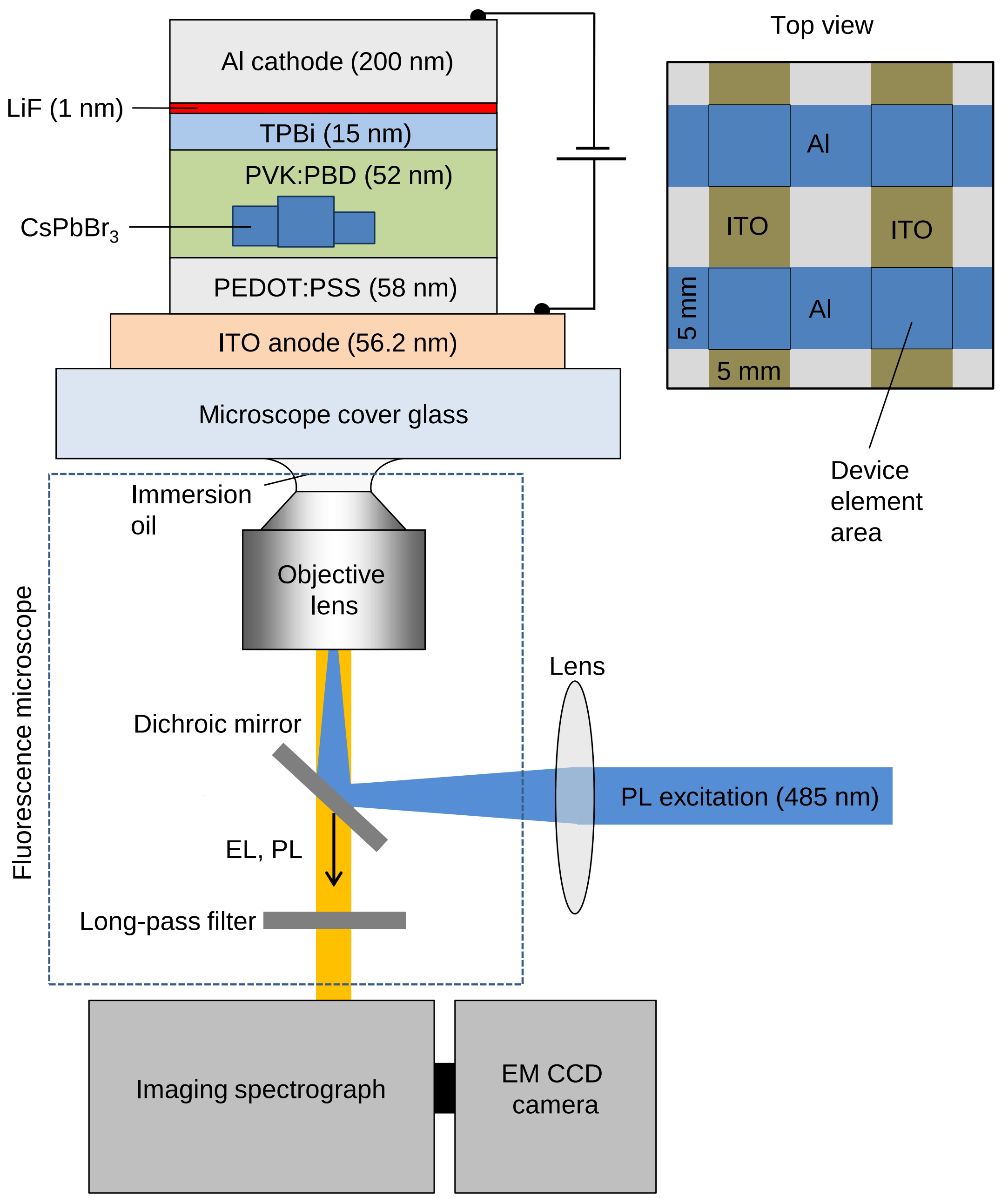

Supplementary Figure 2. Detailed structure of the EL device for single-particle detection, and scheme of the microscopic setup.

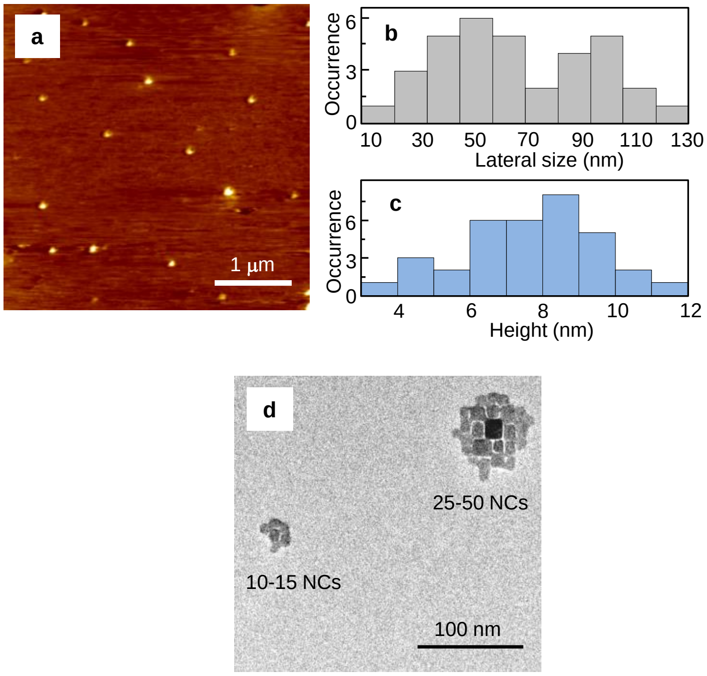

Supplementary Figure 3. a) Atomic force microscopic (AFM) image of aggregates of $CsPbBr_3$ NCs on mica; Distributions of lateral size (b) and height (c) analyzed from the AFM images; d) Transmission electron microscopic (TEM) image of aggregates of $CsPbBr_3$ NCs prepared under the same conditions as in a)

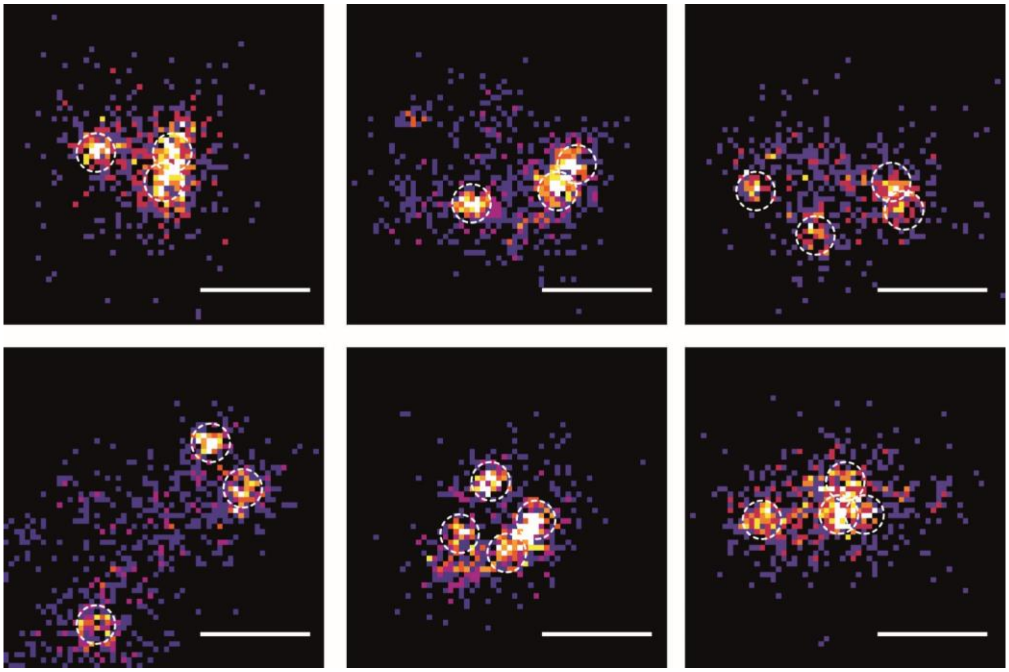

Supplementary Figure 4. Examples of EL super-resolution images of different aggregates at 14 V bias. The scale bar is 100 nm.

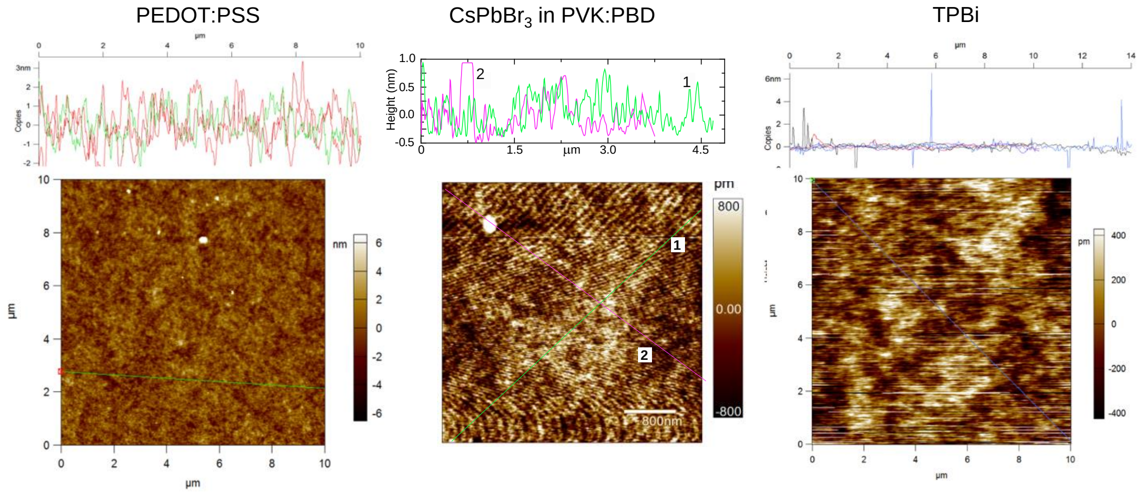

Supplementary Figure 5. AFM images of individual device layers obtained during the device preparation

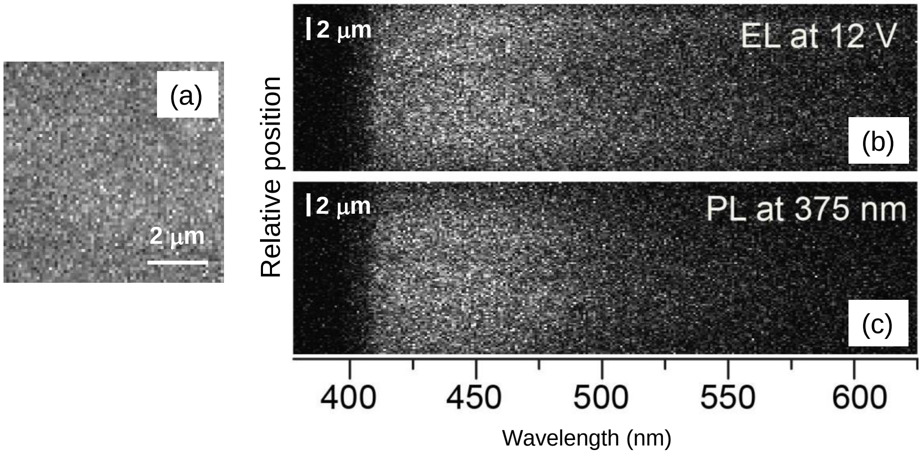

Supplementary Figure 6. Microscopic characterization of an OLED device with a PVK/PBD emitting layer (without doping with perovskite NCs). (a) Microscopic EL image taken at 12 V; spectrally resolved EL (b) and PL (c) image of the same area of the device, showing high degree of device homogeneity and similarity between both excitation modes.

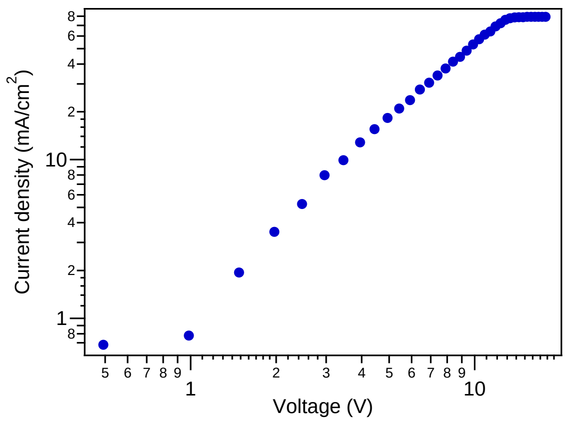

Supplementary Figure 7. I-V characteristics of an ITO / PEDOT:PSS / PVK:PBD / TPBi / LiF / Al device.

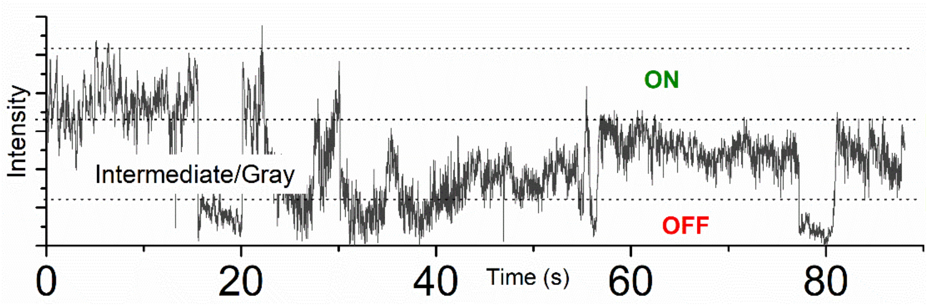

Supplementary Figure 8. Criteria for determining ON, Gray and OFF states for blinking analysis.

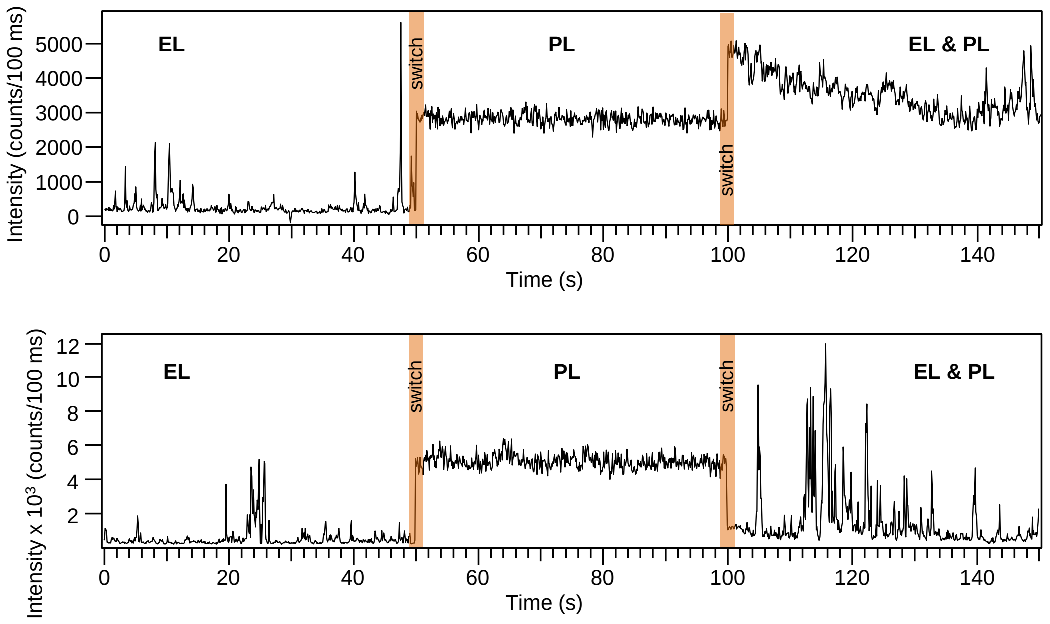

Supplementary Figure 9. Time-traces of emission intensity obtained from two individual aggregates during switching of the excitation modes. PL was excited at 488 nm with 26 W.cm^{-2}, EL with bias of 14 V

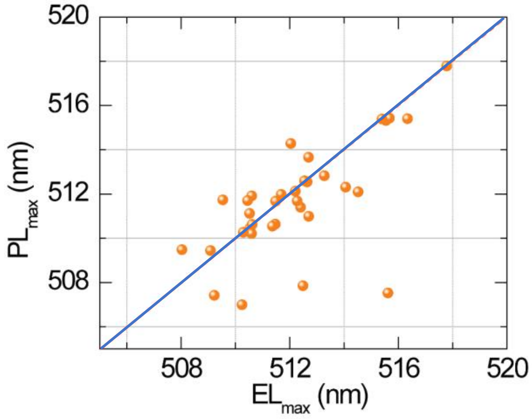

Supplementary Figure 10. Correlation of emission spectral maxima between PL and EL obtained on 37 NCs that were active in both excitation modes.

**Layer**	**Thickness (nm) by AFM**	**Roughness rms (nm)**	**Preparation condition**
ITO coated glass	56.2	-	as received
PEDOT:PSS	58 ± 6	1.162	spin coated as received, filtered by 0.45 μM
PVK:PBD:$CsPbBr_3$	52 ± 10	0.270	1 wt % in toluene spin coated in two steps at 1000 rpm and 4000 rpm for 30s
TPBi	15 ± 5	0.510	vapor deposition
LiF	1 ± 0.2 (expected, optimized for 20 nm)	2.13 nm (for ~11 nm thick film)	vapor deposition
Al	200 ± 10		vapor deposition

Supplementary Table 1. Preparation and characterization of individual layers of the light-emitting device

Supplementary Note 1

Spectroscopic characterization of $CsPbBr_3$ NCs in bulk solutions

UV-vis spectra of $CsPbBr_3$ NCs in toluene with 375 nm excitation show characteristic emission at ~ 516 nm (FWHM ~ 20 nm), comparable to prior reports (Supplementary Figure 1a). The size distributions of NCs obtained from transmission electron microscopy (TEM) show approximately cube-shaped structures with edge lengths of ~ 16 ± 5 nm (Supplementary Figure 1b) indicating broader size distribution as compared to NCs reported for synthesis at ≤ 200 ^{o}C, probably due to the high temperature (225 ^{o}C) set to synthesize slightly larger high-quality $CsPbBr_3$ NCs. We noticed however, that the PL emission width for these NCs synthesized at 225 ^{o}C remains comparable (~ 20 nm) to the $CsPbBr_3$ synthesized at ~200 ^{o}C which can be attributed to the moderate quantum confined effect (with the excitonic Bohr diameter of 7 ~ 11 nm) for these NCs. Comparison of the PL spectra and size distributions of the samples synthesized at ~200 ^{o}C and at ~225 ^{o}C (shown in Supplementary Figure 1d) further confirms the moderate quantum confined effect. Supplementary Figure 1c depicting the excited state lifetime of $CsPbBr_3$ in toluene shows two-exponential relaxation attributed conventionally to trap assisted and excitonic emissions.

Supplementary Note 2

Estimation of the charge recombination rate on a single CsPbBr3 nanocrystal

To estimate the charge recombination rate, we adopted the approach used for imaging of single organic molecules in OLED devices [1]. We assume Langevin-type recombination [2] in which the recombination rate n_r is given by

$$n_r = \frac{\pi r_c^2}{e} j \qquad (1)$$

where j is current density and r_c a capture radius. The capture radius r_c represents a critical distance at which the Coulomb binding energy between an electron and a hole equals the thermal energy kT. The r_c is given by

$$r_c = \frac{e^2}{4\pi\varepsilon kT} \qquad (2)$$

For PVK, the r_c can be estimated as 23 nm. Adding half the NC edge length as a physical dimension of the NC gives an effective r_c value of 31 nm. To determine the current density, we prepared a matrix-only device with the same structure as the one used for the single-particle EL experiments but without the perovskite NCs doped. The current density was determined from an I-V dependence (Supplementary Figure 7) measured on this device. At 14 V, j = 0.08 A.cm^{-2} which gives the recombination rate n_r of 1.2 x 10^{7} s^{-1}. The actual recombination rate will be influenced by many factors and its correct estimation is more complex, and as such the value of n_r obtained above should be treated as an upper limit of the true rate.

Supplementary References:

1. Nothaft, M.; Hoehla, S.; Nicolet, A.; Jelezko, F.; Fruehauf, N.; Pflaum, J.; Wrachtrup, J. Optical Sensing of Current Dynamics in Organic Light-Emitting Devices at the Nanometer Scale. *ChemPhysChem* 12, 2590-2595 (2011)

2. Blom, P. W. M.; de Jong, M. J. M.; Breedijk, S. Temperature dependent electron-hole recombination in polymer light-emitting diodes. Appl. Phys. Lett. **7**1, 930-932 (1997)
